# Irisin, the Myokine: Guardian and Mediator in Cardiovascular System

**DOI:** 10.1002/edm2.70097

**Published:** 2025-11-19

**Authors:** Tian Lan, Xueru Yan, Jie Li, Haoran Gu, Qi Hou, Enpeng He, Qingyuan Yang

**Affiliations:** ^1^ School of Physical Education Xinjiang Normal University Urumqi China; ^2^ Key Laboratory for Monitoring and Evaluation of Combat Sports Training Xinjiang Research Center of Sports Science Urumqi China

**Keywords:** atherosclerosis, cardiovascular, Irisin, stroke

## Abstract

**Background:**

Cardiovascular diseases pose a significant challenge to global health, and the role of exercise as a non‐pharmacological intervention has attracted considerable attention. Irisin, a myokine released during exercise, exhibits excellent potential in regulating metabolism. Its potential intervention value in metabolic and neurodegenerative diseases has been preliminarily confirmed by correlational studies and animal experiments.

**Objective:**

To reveal the unique role of Irisin in the cardiovascular field and clarify its regulatory mechanisms and clinical application prospects in cardiovascular health.

**Methods:**

By comprehensively reviewing existing studies, this paper systematically summarizes the therapeutic effects and molecular mechanisms of Irisin in various cardiovascular diseases (such as atherosclerosis, myocardial infarction, myocardial ischemia‐reperfusion injury, and heart failure) and cerebrovascular diseases (including ischemic stroke, hemorrhagic stroke, and post‐stroke depression), and further explores its association with perivascular adipose tissue.

**Results/Content:**

Irisin demonstrates multi‐dimensional therapeutic potential in the aforementioned cardiovascular and cerebrovascular diseases. Its mechanisms of action involve multiple aspects such as metabolic regulation, inflammation inhibition, and tissue repair. Additionally, it has a close mutual regulatory relationship with perivascular adipose tissue, collectively forming a complex regulatory network for cardiovascular health.

**Conclusion:**

This review provides a theoretical basis for the clinical application of Irisin in cardiovascular diseases, not only opening up new research and application directions but also further highlighting the unique significance of exercise and Irisin in maintaining cardiovascular health.

## Graphical abstract

This graphical abstract illustrates the interconnections between key cardiovascular and cerebrovascular conditions, along with potential influencing factors. Central to the diagram are several critical pathological states: Atherosclerosis, which is linked to downstream cardiovascular events such as myocardial infarction and myocardial ischemia–reperfusion. These cardiac events may progress to heart failure. Additionally, the diagram includes cerebrovascular conditions: Ischaemic stroke and haemorrhagic stroke, which share vascular origins with the aforementioned cardiovascular conditions. Notably, exercise and Irisin are highlighted as potential modulators, suggesting their role in influencing these pathological processes—likely through mechanisms that impact vascular health, inflammation or metabolic regulation.

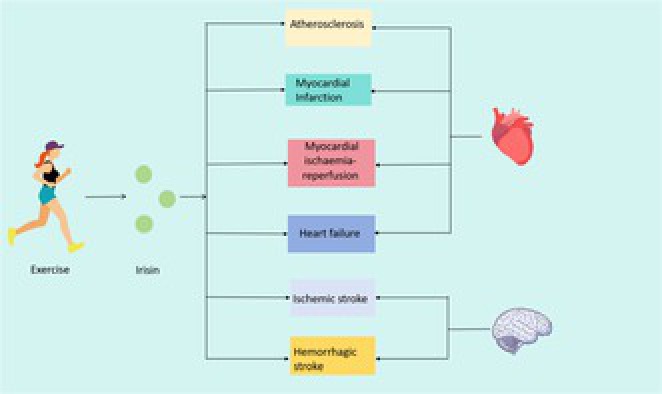

## 1 Introduction

Cardiovascular–cerebrovascular disease (CCD) poses a significant challenge to human health. Globally, it stands as the foremost cause of morbidity, disability and mortality [1]. CCDs represented by coronary heart disease and stroke share a common pathological basis of vascular dysfunction. Emerging evidence has highlighted the critical role of energy metabolism disorders in this pathological process [2, 3, 4]. As a recently discovered regulator of energy metabolism, Irisin has shown potential in vascular protection by modulating mitochondrial function, oxidative stress and inflammatory signalling.

In recent years, exercise has been fervently advocated as a substantial non‐pharmacological strategy for preventing cardiovascular disease. It is regarded as a primary preventive measure that decelerates cardiovascular ageing and promotes longevity [5]. From a macroscopic perspective, healthcare costs are notably lower for cardiovascular disease patients who engage in regular physical activity compared to the non‐exercising population [6]. At a more microscopic level, exercise can inhibit vascular endothelial dysfunction by decreasing levels of soluble intercellular adhesion molecule‐1 (sICAM‐1) [7]. Additionally, therapeutic approaches aimed at rectifying the imbalance of energy metabolism in mitochondria can effectively bolster the neuroprotection of stroke patients [8].

Irisin, a myokine secreted during exercise, plays a crucial role in regulating metabolic disorders. Its initial discovery occurred in studies focused on white fat browning [9]. Recent studies indicate that Irisin is implicated in a range of metabolic and neurodegenerative diseases, extending beyond white fat browning [10, 11, 12, 13]. Furthermore, Zhao's study suggests that exercise‐induced Irisin plays a crucial role in maintaining cardiovascular health through its contribution to angiogenesis. This potential renders it a novel therapeutic target for ischaemic diseases [14]. Similarly, a population‐controlled study has demonstrated an association between lower circulating Irisin levels and higher levels of comorbid cardiovascular disease [15]. Overall, Irisin serves as a connection between exercise and the amelioration of cardiovascular disease, potentially playing a role in pathomechanisms and therapeutic options. For this reason, this paper summarises the therapeutic tools and corresponding mechanisms of Irisin in cardiovascular and cerebrovascular diseases.

## 2 Structure and Biological Function of Irisin

### 2.1 Structural Characterisation and Conservation of Irisin

Irisin was initially discovered by Boström during a study on the browning of white fat and was named after the Greek goddess of the rainbow, Iris. This factor is produced by the cleavage of the precursor protein FNDC5, regulated by PGC‐1α [9]. It is interesting to note that FNDC5 mRNA in humans has a different translation start codon compared to that in experimental mice. Specifically, it has ATA as the translation initiation codon, which is less efficient in translation compared to ATG [16]. It is worth noting that Raschke questioned whether the ATA start codon could efficiently produce Irisin protein, but subsequent studies have shown that this mutation remains the main translation mode of human FNDC5 [17]. Structurally, the FNDC5 protein comprises 209 amino acid residues, including a signal peptide (28aa), an FNIII structural domain (93aa), a linker peptide (30aa), a hydrophobic transmembrane structural domain (19aa) and an intracellular structural domain (39aa). It is worth noting that the signal peptide in humans is 31aa, resulting in the FNDC5 protein having 212 amino acid residues [18]. The Irisin protein consists of 112 amino acid residues, including the FNIII structural domain (93 aa) and part of the transmembrane linker peptide (19 aa).

The molecular weights of FNDC5 proteins vary in different tissues, possibly attributed to variations in the number of attached oligosaccharides during glycosylation modifications [19]. Similarly, Irisin, the secreted segment of the FNDC5 protein, possesses two N‐glycosylation sites, namely, Asn‐7 and Asn‐52 [20]. Nie et al. concluded that the absence of N‐glycosylation in FNDC5 could enhance the incidence of structural instability in the protein and reduce the effective secretion of Irisin [21]. Consequently, the molecular weight of Irisin ranges from approximately 12 to 35 kDa [21]. X‐ray crystallography reveals that the crystal structure of Irisin closely resembles that of the FNIII protein fold. However, in contrast to the FNIII protein fold, the Irisin fold lacks association with glycosylation and forms a continuous intersubunit β‐sheet dimer [18]. This type of dimer implies that Irisin is likely to induce spontaneous signalling at the cell surface [18].

It is noteworthy that the amino acid sequence of FNDC5/Irisin is highly conserved, with the mouse Irisin sequence being fully consistent with the human Irisin sequence [18]. This highly conserved sequence is believed to be associated with the maintenance of basic life functions.

**FIGURE 1:**
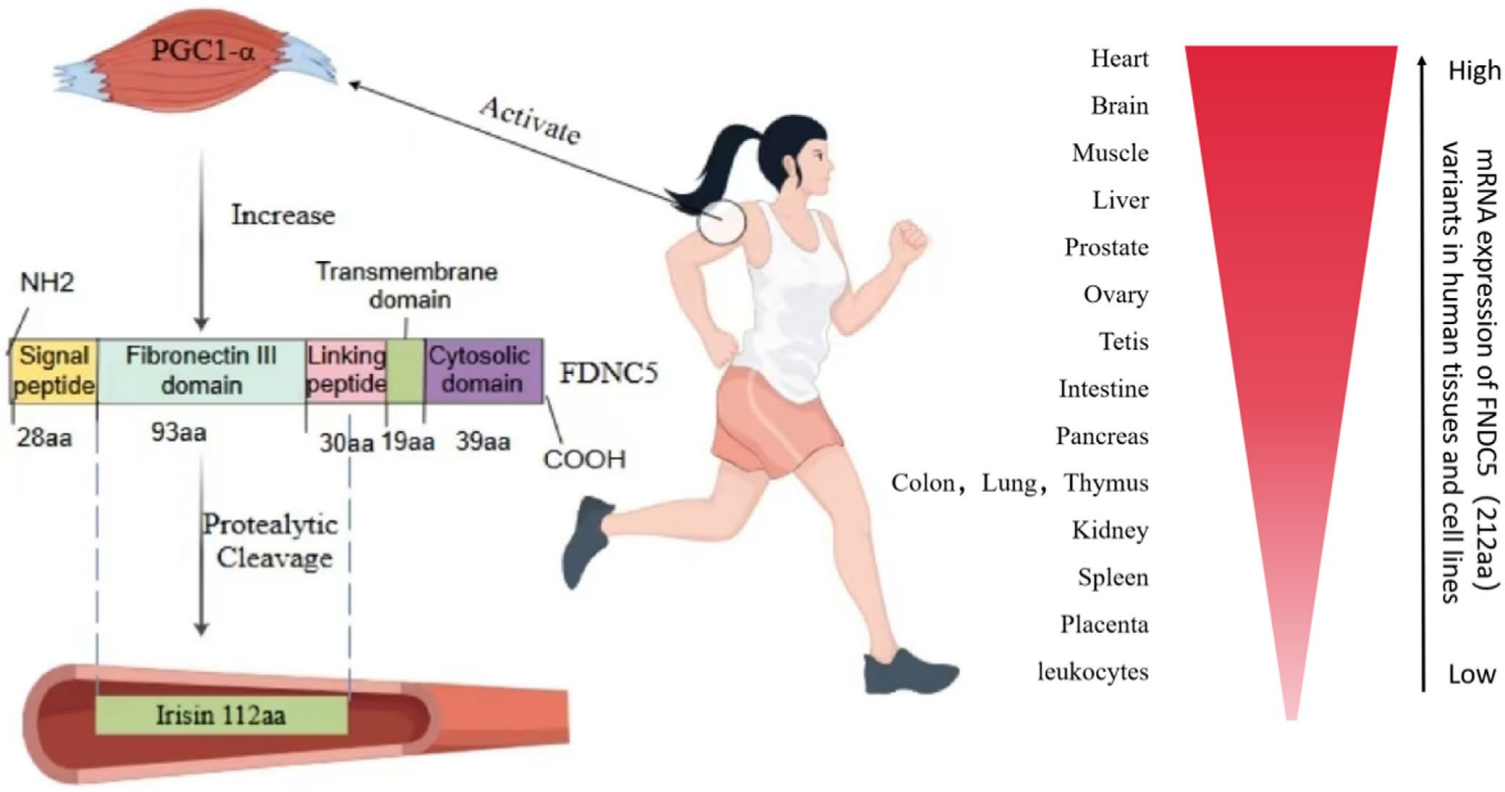
Irisin synthesis via FNDC5 proteolysis and tissue‐specific expression levels. Exercise is the main way to produce Irisin, in which the FNDC5 protein in the human body possesses 212 amino acid residues. In contrast, the Irisin protein, which consists of the FNIII structural domain (93aa) and part of the transmembrane junctional peptide (19aa), has a total of 112aa amino acid residues. The amount of FNDC5 varies from tissue to tissue, and Kim's study was the first to sequence FNDC5 in 16 different cells and tissues [23].

As mentioned by Flori [22] in his article, FNDC5 mRNA is widely expressed in various regions of the brain and muscle (heart and bone). However, in humans, brown adipose tissue, prostate, intestine, pancreas and liver show moderate levels of expression. In rats, FNDC5 mRNA levels are very low or undetectable in white adipose tissue, lungs, kidneys, thymus, spleen, placenta, stomach and liver (Figure 1). We can see that, although the sequence of Irisin is highly conserved in the two organisms, the expression of FNDC5 mRNA, the precursor protein of Irisin, is not the same. The secretion of Irisin is a systemic process, and it is difficult to detect the levels in individual tissues. However, it is possible to look at FNDC5 mRNA expression levels by culturing tissues and cell lines [23]. The current study differs from the initial study in that Irisin is now widely studied not only as a factor related to fat browning and thermogenesis but also as a factor underlying energy metabolism in the human body. Consequently, Irisin is now extensively studied not only as a factor associated with fat browning and thermogenesis but also as a factor underlying energy metabolism in the human body.

### 2.2 Mode of Secretion of Irisin

**FIGURE 2:**
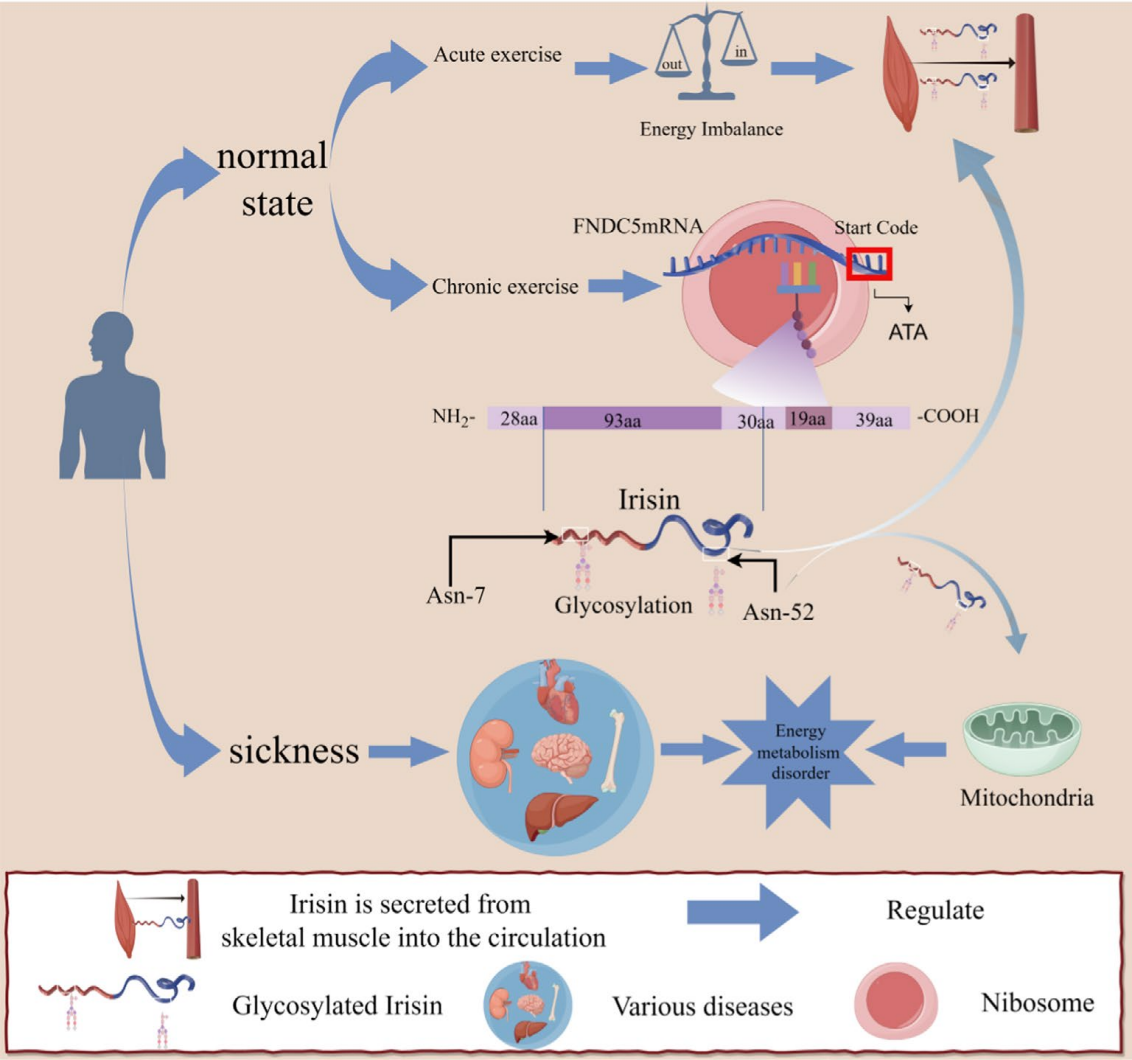
Two modes of Irisin cleavage. In the normal physiological state, there are two distinct patterns that promote the generation of Irisin. First, chronic exercise increases the level of FNDC5 mRNA, with its transcription initiation codon being ATA. This process is associated with an increase in FNDC5 expression, which in turn enhances the production of Irisin. Second, acute exercise disrupts the balance between energy input and output in the body, triggering the proteolytic cleavage of FNDC5 to generate Irisin. In the context of disease states, Irisin plays a pivotal role in ameliorating mitochondrial dysfunction and regulating energy imbalances within various diseased tissues. Its actions are aimed at restoring homeostasis and promoting tissue health. The figure was created using Figdraw.

The currently more plausible hypothesis within the scientific community regarding how exercise releases Irisin is based on Bao's view. According to this perspective, acute exercise releases Irisin into the bloodstream by promoting the cleavage of FNDC5 in skeletal muscle. In contrast, chronic exercise increases the amount of FNDC5 mRNA in tissues [24]. We summarised the two modes of operation of Irisin based on the available evidence to elucidate the relationship between exercise and Irisin in Figure 2.

Under normal conditions, the elevation of circulating Irisin during acute exercise is intended to counteract the stress molecules produced by the exercise. In acute exercise, the body experiences stress, disrupting internal environmental homeostasis and energy balance [25, 26]. In this process, FNDC5 in the muscle is cleaved to release Irisin into the bloodstream. During acute exercise, FNDC5 in the muscle is cleaved, releasing Irisin into the bloodstream. It reaches the organs, activates factors responsible for energy metabolism (such as AMPK and SIRT) and plays a role in mitochondria to rapidly fill the ‘energy gap’ [27, 28]. Chronic exercise differs from acute exercise solely in the accumulation of FNDC5 mRNA, the precursor to Irisin [24]. It is important to note that the accumulation of FNDC5 mRNA is not confined to muscle tissue but is also present in other tissues, such as the myocardium. The amount of Irisin cleaved during a single acute exercise session depends on the amount accumulated during chronic exercise [29, 30, 31, 32].

In a diseased state, Irisin functions as a mitochondrial repair agent. Mitochondrial dysfunction, induced by disease, is the main contributor to the inhibition of energy metabolism in organs. Here, FNDC5, accumulated due to chronic exercise, initiates the cleavage of Irisin. This process enables Irisin to enter the bloodstream and circulate through the diseased organ, repairing the mitochondria. Irisin promotes mitochondrial kinetic homeostasis [33], sustains autophagy [34] and ameliorates the oxidative stress environment [35], contributing to the repair of damaged mitochondria.

### 2.3 Irisin's Multiple Functions

**FIGURE 3:**
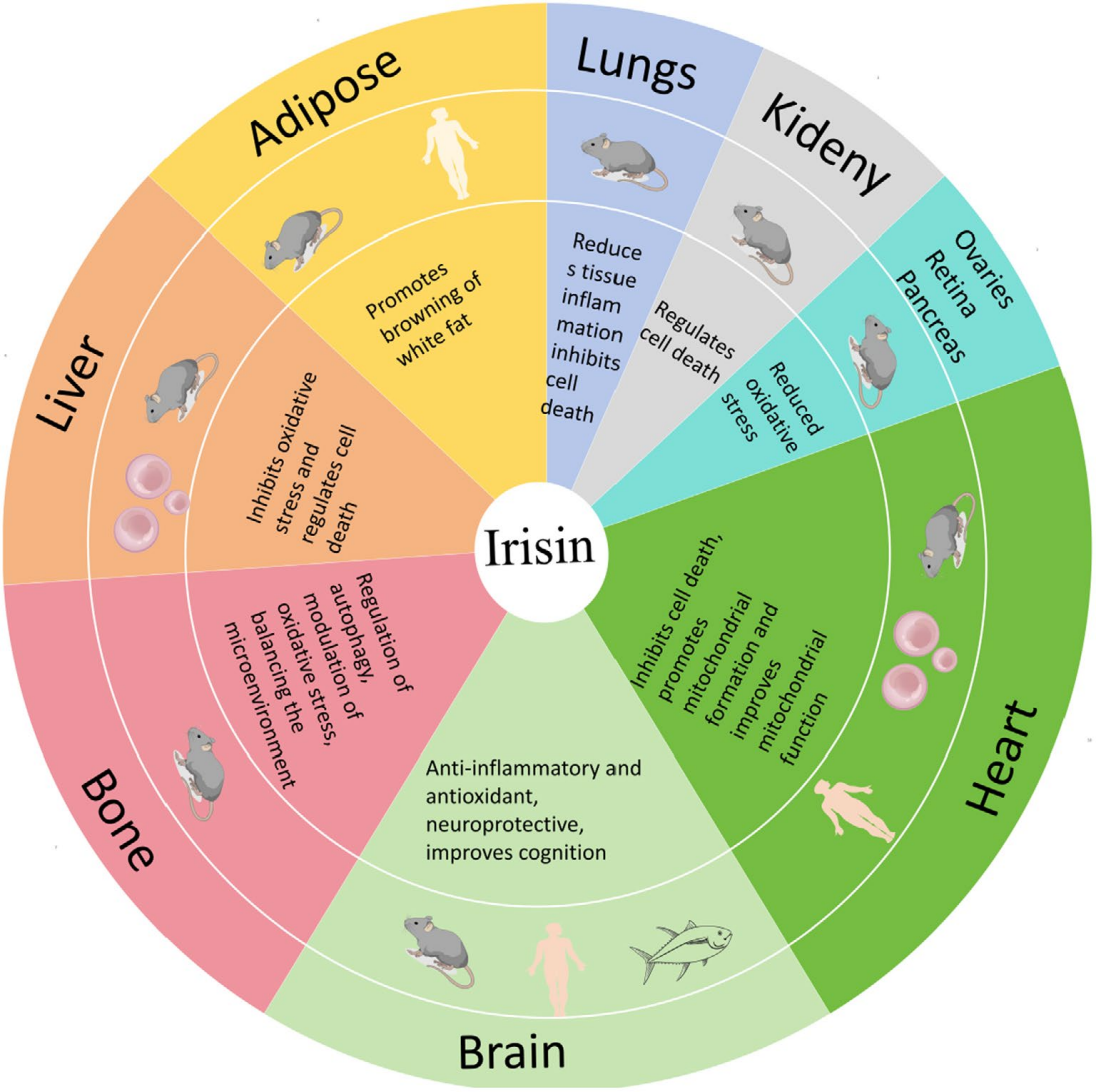
Role of Irisin in various tissues and experimental subjects. Irisin has been studied in various tissues; this figure shows the role of Irisin in each tissue, and it can be seen that Irisin has been studied in the heart and the brain for a large part of the study.

PGC‐1α is a crucial pathway in energy metabolism, playing a significant role in regulating oxidative stress [36, 37]. In cardiac metabolism, it acts as an energy regulator and plays a vital role in cardioprotection [38]. Notably, Irisin regulated by PGC‐1α maintains energy homeostasis by modulating mitochondrial biogenesis and oxidative stress, a mechanism fully evidenced in its involvement in fat browning and cardioprotection [9, 38]. In addition to its initially identified role in fat browning [39, 40, 41, 42, 43], Irisin is involved in bone remodelling through αVβ5 [44] and in maintaining glucose homeostasis in the liver [45]. Furthermore, Irisin has a unique role in the nervous system [46, 47, 48, 49, 50, 51, 52, 53], upregulating the expression level of the neurotrophic factor BDNF and promoting synaptic plasticity [54, 55]. Therefore, Bao suggests that Irisin could be an ideal therapeutic target for both metabolic and non‐metabolic diseases [24]. Current research has demonstrated the therapeutic effects of Irisin on other organs (lungs [56, 57, 58], liver [45, 59, 60, 61, 62, 63], bones [64, 65, 66, 67, 68, 69, 70], ovaries [71], kidneys [72, 73, 74], retina [75], heart [30, 31, 76, 77, 78, 79, 80, 81, 82, 83]), as well as pancreas [84] (Figure 3).

Irisin's association with mitochondria has led to claims that it can cure a range of diseases and conditions. Recent studies have shown that Irisin activates gene expression related to mitochondrial fusion and improves mitochondrial kinetic dysfunction [85, 86]. In cases of myocardial injury, Irisin intervention has been shown to improve cardiac dysfunction caused by ischaemia and hypoxia by modulating the ferroptosis pathway and the mitochondrial ubiquitin ligase mechanism [87, 88]. The heart is a vital organ in the human body, and as a result, it consumes a significant amount of energy. This is reflected in the higher number of mitochondria present in the heart compared to other organs [89]. Critically, although Irisin is released in various organ tissues, circulating Irisin is mainly supplied by cardiac and skeletal muscle [90, 91, 92]. The large number of mitochondria in the myocardium and the presence of Irisin have led the scientific community to believe that Irisin maintains mitochondrial homeostasis, regulating energy metabolism in the heart.

## 3 Mechanisms and Effects of Irisin in the Treatment of Cardiovascular and Cerebrovascular Diseases

### 3.1 Irisin and Coronary Artery Disease

Widely recognised as a crucial factor in improving cardiorespiratory fitness, aerobic exercise deserves consideration regarding the role of exercise‐secreted Irisin. Data from Japan indicate a correlation between circulating Irisin levels and various indicators of health assessment. In a cross‐sectional study involving 328 Japanese citizens, Inoue discovered a negative correlation between circulating Irisin levels and cardiometabolic risk scores, irrespective of gender and age [93]. This finding implies that Irisin may play a significant role in preventing heart disease, aligning with the established benefits of exercise in enhancing cardiorespiratory function. Alipoor presents a different perspective in another cross‐sectional study; this research, which recorded Gensini scores in 166 adults, indicated that circulating Irisin was not significantly associated with the likelihood of developing coronary artery disease [94]. The discrepancy is attributed to variations in research methodology, and according to Ou‐Yang, the inconsistency in cross‐sectional study results is linked to differences in the level of measurement [95]. Despite the absence of suitable measurement methods, independent studies have demonstrated a promising therapeutic effect of Irisin on coronary artery disease.

#### 3.1.1 Atherosclerosis

Atherosclerosis (AS) is a complex arterial pathology characterised by systemic inflammation and the aggregation of plaques within the arterial wall. It stands as the primary contributor to myocardial infarction and stroke events [96, 97]. The investigation into the relationship between Irisin and AS traces back to Sesti's 2014 cross‐sectional study involving 192 white adults. The statistical analysis of the study unveiled a positive association between Irisin and intima‐media thickness (IMT), suggesting that elevated levels of circulating Irisin were associated with an increased incidence of AS [98]. However, recent studies have contradicted Sesti's findings, suggesting that Irisin levels are lower in patients with AS [99, 100]. Notably, two independent studies on subclinical AS in leukaemia have reported similar findings [101, 102]. In patients with this systemic inflammatory disease, serum Irisin levels decrease, and there is an increase in IMT [101, 102]. Additionally, patients with mid‐axial spondyloarthritis exhibit accelerated atherosclerosis in clinical settings, with low Irisin levels hastening plaque formation and, consequently, demonstrating a strong correlation with the presence of plaques [103]. Recent evidence suggests that the association between Irisin and AS may be mediated through thyroid function and may involve bidirectional regulatory mechanisms ([104]). Specifically, thyroid dysfunction is thought to directly or indirectly influence the regulation of Irisin, and in turn, Irisin may have a regulatory effect on thyroid activity. Of note, clinical hypothyroidism and subclinical hypothyroidism (SCH) have been identified as independent risk factors for the pathogenesis of atherosclerosis and cardiovascular disease [105], but the currently available data on the relationship between thyroid function and Irisin are scarce and present the same problems as described previously. Although the results of the earlier studies are quite different from the more recent ones, these studies are pointing to an expectation that Irisin can be used as an early predictive biomarker for AS. However, the use of this biomarker has to be demonstrated by more factual evidence of its relevance. In other words, its specific association with AS markers such as IMT needs to be demonstrated.

**FIGURE 4:**
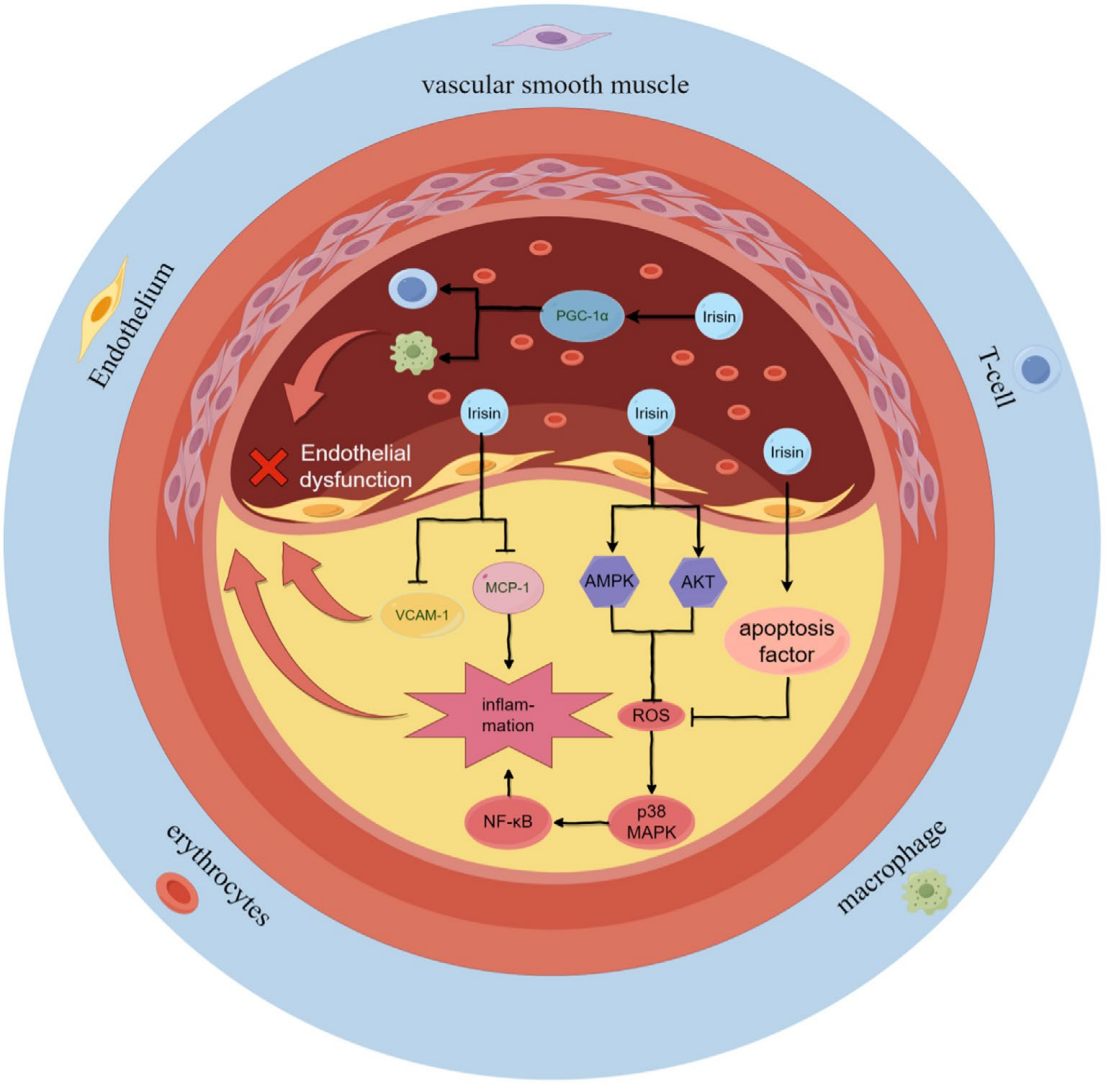
The role and mechanism played by Irisin in atherosclerosis. The role of Irisin in atherosclerosis is multifaceted and involves several key mechanisms. First, Irisin regulates VCAM‐1 to initiate leucocyte adhesion and MCP‐1 to mediate their migration, jointly suppressing the inflammatory cascade to inhibit vascular inflammation and improve endothelial dysfunction. Second, in terms of oxidative stress, Irisin activates the AMPK and AKT pathways, leading to a reduction in ROS generation. Finally, in relation to cell apoptosis, Irisin modulates apoptotic factors and suppresses the ROS/p38 MAPK/NF‐κB signalling pathway. The figure was created using Figdraw.

Investigating the intrinsic mechanisms through both in vivo and in vitro studies will enhance the credibility of Irisin as a biomarker for AS, as described above. APOE knockout mice with FNDC5 overexpression exhibited a significant reduction in the area of aortic plaque [106]. This outcome was attributed to the treatment of endothelial dysfunction and the suppression of vascular inflammation. In Lu's study, Irisin treatment led to a reduction in the expression of inflammatory factors, including macrophages and T lymphocytes. Additionally, it significantly improved endothelial dysfunction by reducing the apoptosis of endothelial cells, compared to AS mice treated with saline only [107]. Similarly, Shimba demonstrated that the Irisin‐dependent protein PGC‐1α has the ability to decrease the expression levels of VCAM‐1 and MCP‐1, reduce cellular accumulation on vascular endothelial cells and inhibit vascular inflammation [108]. Although animal models can offer valuable information about AS, a mechanistic understanding of the condition necessitates evidence from relevant in vitro models [109]. Results from in vitro experiments revealed that Irisin inhibited apoptosis in HUVECs (umbilical vein endothelial cells) and promoted the phosphorylation of AMPK and AKT, leading to reduced oxidative stress production [107]. Building upon this foundation, Zhang conducted an in‐depth study, and the experimental results indicated that Irisin modulated certain apoptotic factors (Bcl‐2, Bax and caspase‐3) to reduce apoptosis. Moreover, it inhibited the expression of inflammatory genes by suppressing the ROS/p38 MAPK/NF‐κB signalling pathway, thereby inhibiting the expression of inflammatory genes [110]. Additionally, in vitro, Zhang's study unveiled another regulatory pathway, indicating that Irisin may also regulate the Akt/mTOR/Nrf2 pathway. This regulatory mechanism could potentially aid in attenuating oxLDL‐induced vascular injury [111] (Figure 4).

#### 3.1.2 Myocardial Infarction

Myocardial infarction (MI) is a significant cardiovascular event resulting from persistent ischaemia caused by the occlusion of coronary arteries, leading to myocardial injury and necrosis [112]. Exercise is widely recognised as a protective factor against cardiovascular events. However, the relationship between exercise and MI is multifaceted. Vigorous physical activity elevates the risk of myocardial infarction in individuals who do not engage in regular exercise [113]. Conversely, moderate and regular physical activity demonstrates a more favourable cardioprotective effect [114]. Irisin elucidates this phenomenon at the molecular level.

The relationship between myocardial Irisin levels and adverse cardiovascular outcomes has garnered significant attention in research exploring the connection between exercise and MI. In one of his 3‐year follow‐up studies, Xie observed that an increase in serum Irisin concentrations was associated with an elevated risk of adverse cardiovascular outcomes after MI [115]. To investigate the role of Irisin in the myocardium, the researcher conducted animal experiments. Mice were implanted with adenovirus‐vectored Irisin, leading to enhanced myocardial mitochondrial respiration, increased oxygen consumption rate and elevated reactive oxygen species production [80]. Both clinical and animal studies suggest that elevated Irisin concentrations are counterproductive in the treatment of MI. Notably, excluding these two studies from the same team, more studies have demonstrated that low Irisin levels are associated with adverse cardiovascular outcomes. In 2014, Emanuele compared serum Irisin levels measured in healthy centenarians, healthy young adults and patients with MI. Her results indicated that young patients with MI exhibited the lowest serum Irisin levels, even lower than those of centenarians [116]. A similar follow‐up study on adverse cardiovascular events after MI, in contrast to Xie's study, revealed that low serum Irisin levels were significantly associated with such events [117]. Additionally, circulating Irisin levels were linked to a high degree of vascular stenosis in patients with MI [118]. Consequently, Irisin has been recognised in recent years as a novel biomarker for predicting MI, comparable to CK‐MB [118, 119, 120].

**FIGURE 5:**
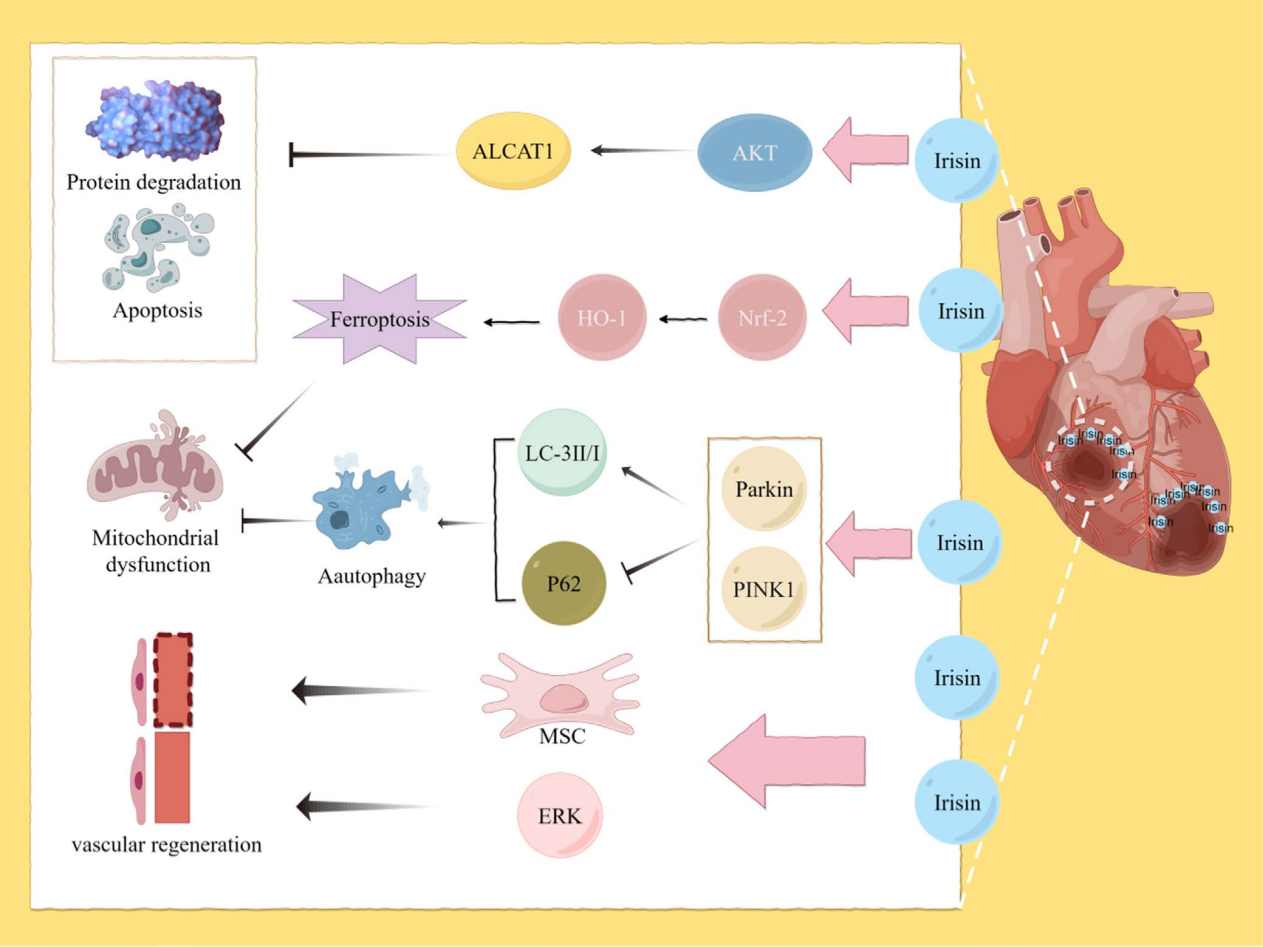
The role and mechanism played by Irisin in myocardial infarction. The role of Irisin in myocardial infarction is mediated through several distinct mechanisms. First, Irisin suppresses the expression of ALCAT1 factor by activating the Akt signalling pathway, thereby mitigating protein degradation and cellular apoptosis. Second, Irisin activates the Nrf2/HO‐1 pathway, leading to reduced ferroptosis and attenuated mitochondrial damage. Additionally, Irisin enhances mitochondrial autophagy through the activation of the PINK1/Parkin‐LC3/P62 pathway. Finally, Irisin promotes angiogenesis by increasing the number of mesenchymal stem cells (MSCs) and phosphorylating ERK. The figure was created using Figdraw.

Irisin plays a prominent role in contributing to recovery after myocardial infarction by exerting significant effects in anti‐inflammation, antioxidative stress and anti‐apoptosis [121]. Cardiomyocyte loss and energy imbalance during acute MI are closely associated with apoptosis induced by lipotoxicity. Therefore, Moscoso utilised H9C2 cardiomyocytes to examine the protective effects of Irisin in the context of MI. The experimental results demonstrated that Irisin treatment of H9C2 cells counteracted apoptosis induced by lipotoxicity and hypoxia. This effect was closely related to the activation of the Akt pathway [122]. Similarly, Wu's study demonstrated that aerobic exercise after MI increased Irisin expression and decreased ALCAT1 expression, thereby attenuating oxidative stress and apoptosis [123]. ALCAT1, or acylprotein thioesterase 1, is an enzyme that participates in oxidative stress regulation by catalysing the deacylation of palmitoylated proteins, and its abnormally high expression is closely associated with cell apoptosis. Another study supported the same idea that aerobic exercise reversed protein degradation and apoptosis after MI through upregulation of Irisin and inhibition of ALCAT1 [124]. In addition to improving oxidative stress pathways, Irisin may treat MI by enhancing mitochondrial homeostasis. Hypoxia leads to cellular ferroptosis, increased iron metabolism and the onset of mitochondrial dysfunction. In contrast, Irisin‐treated cardiomyocytes exhibited reduced ferroptosis and reversed hypoxia‐induced mitochondrial dysfunction [87]. Cao has explored this mechanistically, suggesting that Irisin activates Nrf2/HO‐1, thereby reducing both ferroptosis and mitochondrial damage [87]. Irisin also plays a distinct role in mitochondrial autophagy. Li demonstrated that resistance to exercise is essential for the activation of the PINK1/Parkin‐LC3/P62 pathway by Irisin/FNDC5, enhancing mitochondrial autophagy [35]. Additionally, it has been suggested that OPA1 plays a role in the Irisin‐regulated autophagy pathway, consistent with Xin's view [125]. Furthermore, Irisin promotes angiogenesis via MSCs for the treatment of MI [126, 127]. Irisin treatment also significantly increased the phosphorylation of ERK, contributing to angiogenesis, and significantly improved both infarct size and myocardial fibrosis after MI [128]. Figure 5 summarises the mechanism of action of Irisin in MI.

#### 3.1.3 Cardiac Ischaemia‐Reperfusion Injury

Following a myocardial infarction, surgical intervention is commonly utilised to achieve myocardial reperfusion. This constitutes the most effective strategy for reducing the size of the infarction and enhancing clinical outcomes. However, the myocardium that has experienced ischaemia is susceptible to ischaemia–reperfusion injury (IR) upon restoration of blood flow [129, 130]. IR impairs the cardiac regulatory response and induces metabolic abnormalities in cardiomyocytes, including oxidative stress, systemic inflammation, mitochondrial homeostasis disorders and an imbalance in iron metabolism [131]. Recent literature has shown that Irisin exerts a beneficial therapeutic effect on IR. IR frequently occurs after vascular perforator flap transplantation, resulting in surgical failure. Zhao administered continuous injections of Irisin into the tail vein of rats for 3 days before vascular clamping of the perforator valve to observe changes in the area of flap survival. The results indicated a significantly larger area of flap survival in rats treated with Irisin compared to control rats, along with a higher density of microvessels [132]. Similarly, Wang's study demonstrated a significant improvement in ventricular function after perfusion in ischaemic hearts pretreated with Irisin [133]. Further investigation into the intrinsic mechanisms by which Irisin improves myocardial IR revealed its main effects in improving mitochondrial dysfunction, regulating endoplasmic reticulum (ER) stress and reducing inflammatory responses.

A study was conducted on H9C2 cardiomyoblasts to investigate the effects of pretreatment with Irisin. The study found that Irisin significantly increased SOD‐1 and p38 phosphorylation, indicating its role in inhibiting oxidative stress and protecting mitochondrial function [133]. This finding is supported by another study conducted by Wang, demonstrating that Irisin intervention restored impaired SOD activity [134]. Interestingly, this study also co‐localised Irisin with mitochondria, and the results suggested a pattern of overlap between Irisin and Cox IV (a mitochondrial marker) [134]. Irisin may improve mitochondrial function by ameliorating oxidative stress pathways and may also be involved in regulation through the autophagy pathway. In a study, rats were pretreated with Irisin for 1 week before undergoing IR surgery, and mitochondrial autophagy proteins were measured 24 h after perfusion. The study found that the group treated with Irisin exhibited a significant increase in the levels of PINK3 and Parkin proteins [135]. In a mouse model of diabetic myocardial ischaemia, Irisin exhibited favourable therapeutic effects. Xin observed that treatment with Irisin improved the compromised AMPK pathway, enhancing mitochondrial potential and promoting mitochondria‐associated cell survival [136]. Additionally, Irisin played a crucial role in alleviating ER stress, a significant pathway in the treatment of myocardial IR. Pretreatment with Irisin resulted in decreased expression of stress‐related proteins in the ER, including GRP78 and CHOP [137]. Lu's experiments also confirmed this, showing that Irisin intervention reduced the already elevated levels of IRE1α protein in IR [88]. In myocardial IR, NLRP3 inflammatory vesicles can trigger endothelial dysfunction. Xin's study demonstrated that Irisin reduced the activation of NLRP3 in diabetic myocardial IR, thereby mitigating cellular damage [136].

**FIGURE 6:**
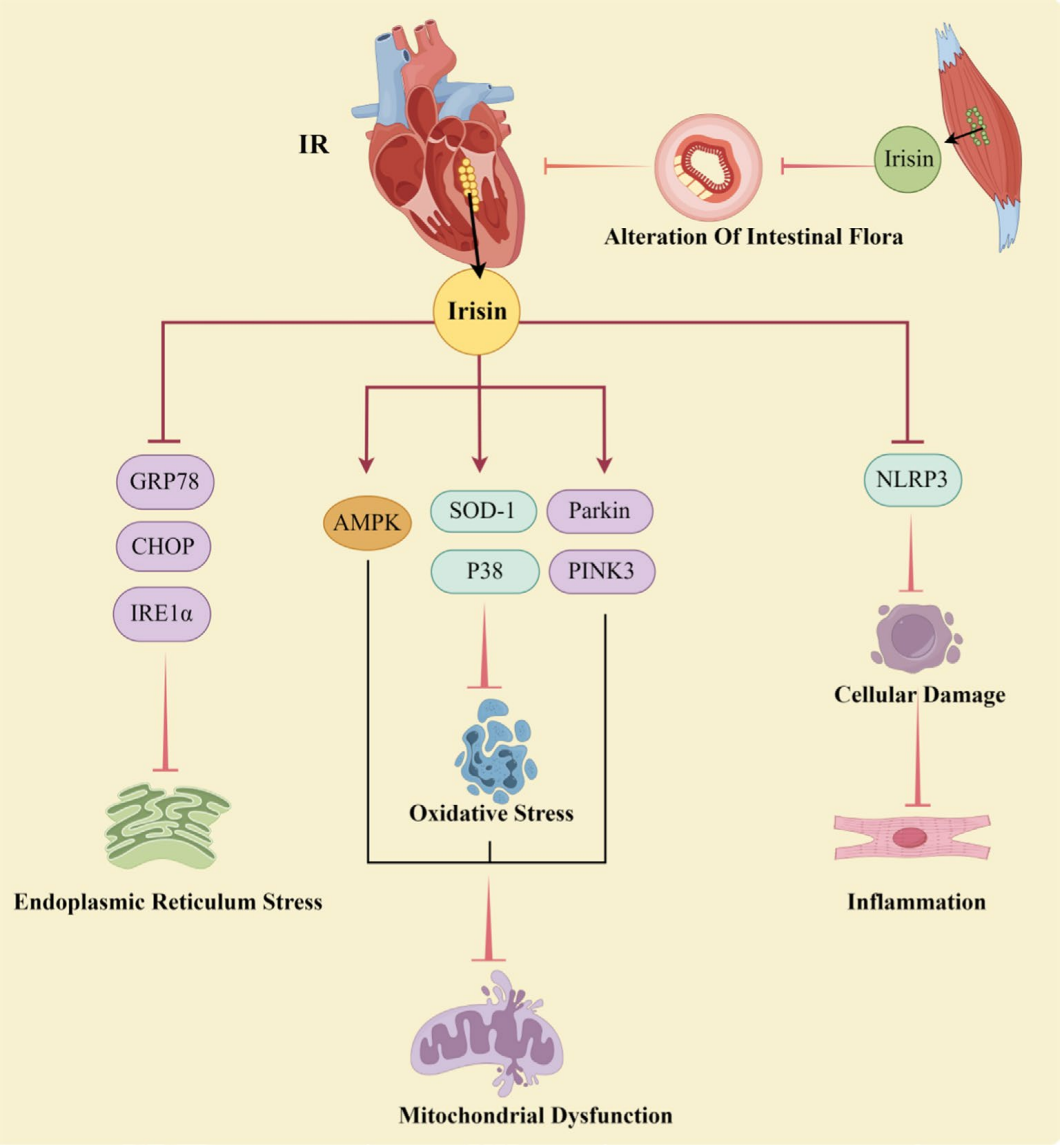
The role and mechanism played by Irisin in myocardial ischaemia–reperfusion. The role of Irisin in myocardial ischemia can be elucidated through three primary mechanisms. First, in ER stress, Irisin suppresses the expression of ER stress‐related proteins, including GRP78, CHOP and IRE1α. Second, in mitochondrial dysfunction, Irisin activates the AMPK pathway, promoting the phosphorylation of SOD‐1 and p38, thus mitigating oxidative stress. Additionally, Irisin upregulates the expression of PINK3 and Parkin proteins, leading to the enhancement of mitophagy. Finally, in cellular inflammation, Irisin inhibits the activation of NLRP3, thereby alleviating inflammation caused by cellular injury. The figure was created using Figdraw.

The above evidence suggests that Irisin may exert beneficial effects in modulating myocardial IR, primarily by preserving mitochondrial function, ameliorating ER stress and alleviating inflammatory pathways (Figure 6). Interestingly, a recent study by Liu investigated the gut–heart axis, revealing a significant association between myocardial IR and gut dysbiosis. Treatment with Irisin reduced the abundance of Actinobacillus flora and increased the abundance of the thick‐walled bacteria phylum, thereby reversing gut dysbiosis to an extent that improved myocardial IR [83].

#### 3.1.4 Heart Failure

Heart failure (HF) is a prevalent end‐stage manifestation of various cardiac diseases, often accompanied by complex comorbidities [138, 139]. Studies indicate that, in developed countries, the prevalence of HF ranges from 1% to 2% [140]. In addition, 34% of hypertensive patients suffer from sarcopenia [140], adult sarcopenic patients have a higher chance of developing chronic heart failure [141] and sarcopenia can also lead to other comorbidities related to non‐alcoholic fatty liver disease, T2DM, obesity, chronic liver disease and cancer, which have been shown to be associated with cardiovascular disease [142]. Could alleviating sarcopenia reduce the impact on cardiovascular disease? Circulating levels of Irisin, a biomarker predictive of sarcopenia, are of research importance [143]. The societal burden of HF is substantial. Energy deficiency emerges as a pivotal factor in HF development and is linked to diminished mitochondrial biogenesis and function [144]. Morphologically, it is evident that mitochondria display abnormal volume numbers and structural integrity loss [145]. Irisin, a pivotal myokine for enhancing energy metabolism, is abundantly present in the myocardium [146]. Consequently, numerous scholars have investigated the correlation between Irisin and HF.

In 2012, Lecker et al. conducted an initial investigation into the correlation between Irisin expression and HF. The study compared the aerobic exercise capacity of individuals with HF and discovered that FNDC5 expression in skeletal muscle was significantly higher in those with elevated aerobic exercise capacity [147]. Following this, Matsuo extended his research by demonstrating a substantial decrease in FNDC5 and Irisin levels in the skeletal muscle of HF rats. He suggested that this reduction may be linked to an increase in inflammatory factors such as TNF‐α and angiotensin II [148]. These trials have significantly influenced the amelioration of exercise intolerance symptoms in patients with heart failure and have provided valuable insights. Crosstalk between peripheral and cardiac organs contributes to responding to the presence or absence of abnormal lesions in cardiac organs. A subsequent investigation revealed that serum Irisin can serve as a predictive biomarker for all‐cause mortality in patients with acute heart failure [149]. Kalkan's study also demonstrated similar findings, revealing significantly higher serum Irisin levels in HF patients with cardiac cachexia compared to controls [150]. However, recent studies have yielded conflicting results. El‐Mottaleb found lower serum Irisin levels in patients with concomitant myocardial infarction and HF [151]. Additionally, the analysis revealed a positive correlation between serum Irisin levels, ejection fraction and HDL‐C [151]. Currently, it has been found that Irisin levels in patients with heart failure are negatively correlated with high troponin I, CK‐MB, tumour necrosis factor (TNF), total cholesterol, low‐density lipoprotein cholesterol and triglyceride levels. In contrast, it is positively correlated with left ventricular ejection fraction (LVEF) and high‐density lipoprotein cholesterol levels [152]. In agreement with this, all three of Berezin's studies on the association between serum Irisin and HF demonstrated that, in patients with chronic HF, acute HF or HF with preserved ejection fraction, circulating Irisin levels were significantly lower in these patients than in the corresponding control group [153, 154, 155]. It is important to note that studies suggesting a positive association between elevated circulating Irisin levels and the onset of HF were published earlier. Irisin is a factor in maintaining energy metabolism homeostasis, as demonstrated in our earlier research. In the later stages of the condition, organs and tissues release Irisin to sustain energy balance, resulting in elevated levels consistent with imaging evidence [156].

**FIGURE 7:**
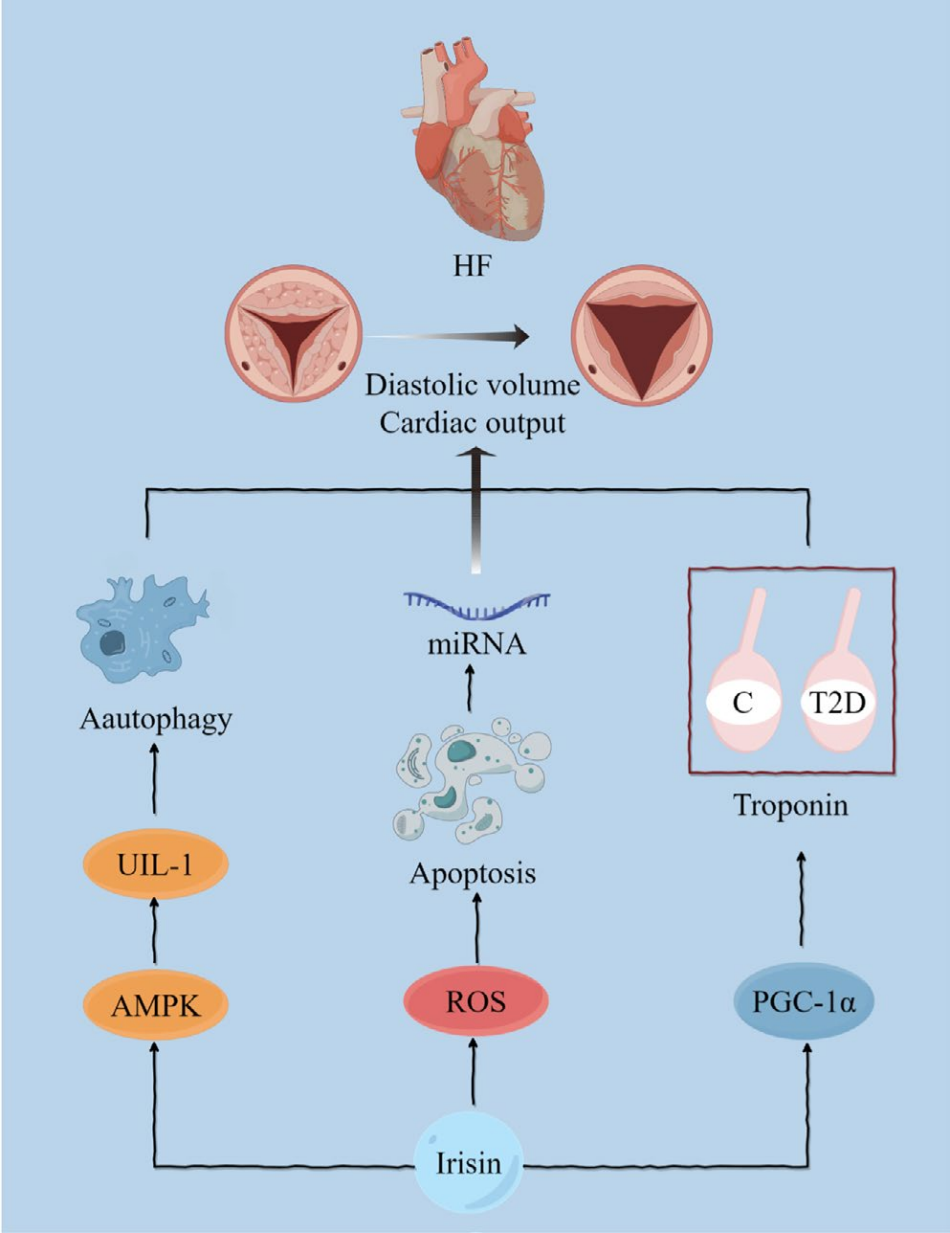
The role and mechanism played by Irisin in heart failure. Irisin plays a pivotal role in augmenting cardiac output in heart failure through three distinct mechanisms. First, Irisin upregulates miRNAs, resulting in the reduction of ROS production and subsequent attenuation of apoptosis. Second, Irisin promotes the upregulation of PGC‐1α, troponin C and troponin T2D, thereby enhancing myocardial function. Finally, Irisin activates the AMPK‐ULK1 signalling pathway, leading to the induction of protective autophagy. The figure was created using Figdraw.

Irisin can function as a biomarker for heart failure and holds potential therapeutic applications. Peng employed H9C2 cardiomyocytes to simulate oxidative stress injury and treated them with Irisin. The results demonstrated that Irisin alleviated apoptosis and reduced reactive oxygen species (ROS) in H9C2 cells. This effect was mediated by the upregulation of miRNAs [157], which play a crucial role in the development of cardiac diseases, including HF [158]. Similarly, at the cellular level, Sundarrajan's study concluded that the knockdown of Irisin in zebrafish results in a reduction in PGC‐1α, troponin C and troponin T2D [159]. The study also evaluated the cardiac function of zebrafish following Irisin intervention. Ultrasound imaging techniques revealed that the administration of Irisin increased the heart's diastolic volume and cardiac output [159]. To observe the protective effect of Irisin on cardiac hypertrophy, Li conducted animal experiments using FNDC5 knockout mice and FNDC5 transgenic mice. The study revealed that the extent of cardiac hypertrophy injury was lower in FNDC5 transgenic mice than in FNDC5 knockout mice [160]. Subsequent studies have demonstrated that the mechanism of cardioprotection by Irisin involves the activation of the AMPK–ULK1 pathway, inducing protective autophagy [161] (Figure 7). Additionally, two population studies have suggested that the protective effect of Irisin is associated with the modulation of oxidative stress [162, 163].

### 3.2 Irisin and Cerebrovascular Injury

**FIGURE 8:**
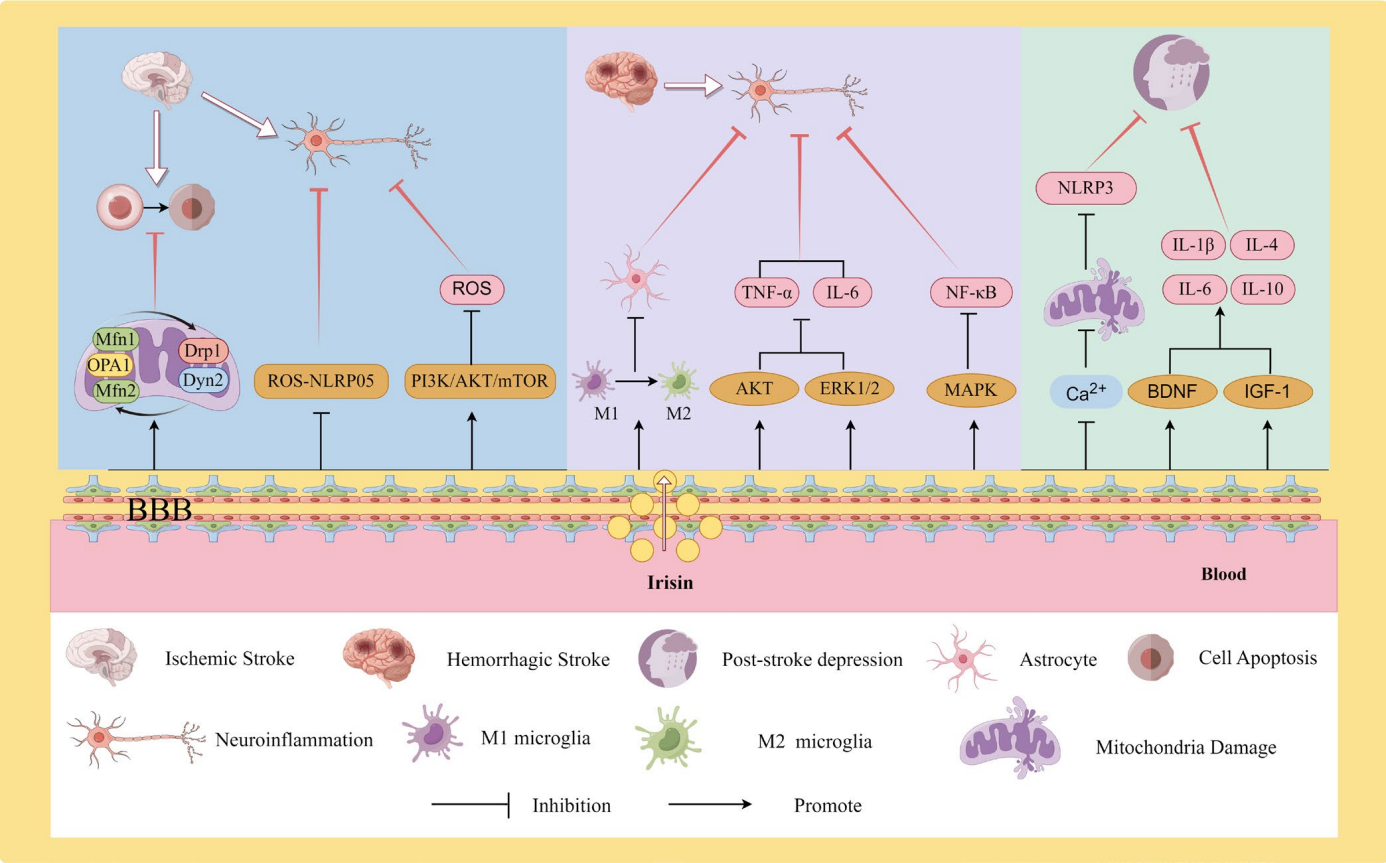
The role and mechanisms played by Irisin in cerebrovascular injury and post‐stroke depression. In ischaemic stroke: (1) Irisin preserves mitochondrial homeostasis by regulating mitochondrial dynamics proteins, thereby suppressing cellular apoptosis. (2) Irisin inhibits ROS‐NLRP3 activation, mitigating neuronal damage. (3) By activating the PI3K/AKT/mTOR signalling pathway, Irisin attenuates ROS production and alleviates oxidative stress‐induced neuronal injury. In haemorrhagic stroke: (1) Irisin promotes the transition of microglia from the M1 phenotype to the M2 phenotype, suppressing inflammatory responses triggered by astrocyte activation. (2) Irisin modulates the phosphorylation of AKT and ERK1/2, resulting in the downregulation of TNF‐α and IL‐6 mRNA expression. (3) Additionally, Irisin inhibits the activation of the MAPK pathway, reducing NF‐κB activation. In post‐stroke depression: (1) Irisin enhances the expression of brain‐derived neurotrophic factor (BDNF) and insulin‐like growth factor‐1 (IGF‐1) in the brain, suppressing inflammatory signalling molecules. (2) Irisin also ameliorates mitochondrial damage, thereby blocking NLRP3 inflammatory responses. The figure was created using Figdraw.

Cerebrovascular disease, commonly referred to as ‘stroke’, manifests as a clinical syndrome characterised by the deprivation of blood flow to the brain due to the occlusion or rupture of cerebral blood vessels [164]. Consequently, strokes are categorised as either ischemic or haemorrhagic [165]. Irisin, a novel myokine, has garnered significant attention in neuroscience research over recent years, particularly in the context of diseases like Alzheimer's disease and diabetic mild cognitive impairment (Figure 8).

#### 3.2.1 Ischemic Stroke

According to Tu's statement following a comprehensive long‐term follow‐up study involving 1530 Chinese patients with ischemic stroke [166], Irisin emerges as an independent prognostic marker for ischemic stroke. The study sought to assess the predictive value of serum Irisin levels and NIHSS stroke scores concerning functional outcomes and mortality in patients with ischemic stroke [166]. Similar conclusions were drawn in Wu's study involving Chinese ischemic stroke patients [167]. In a separate observational study conducted in Japan, researchers explored the relationship between serum Irisin levels and cerebral small vessel disease, a condition associated with stroke. The findings indicated that elevated Irisin levels were linked to a diminished burden of cerebral small vessel disease in healthy men and hinted at the potential for intraluminal infarction [168]. These clinical studies underscore the potential of Irisin levels as a novel and dependable prognostic factor for ischemic stroke.

Irisin serves not only as a prognostic factor in ischemic stroke but also exhibits protective and therapeutic effects on ischemic brain tissue. Nerve cells are highly susceptible to changes in energy supply during cerebral ischemia, leading to a cascade of pathophysiological processes [169]. The presence of Irisin has been demonstrated to exert a protective effect on nerve cells. In the MCAO mouse model, Irisin treatment significantly decreased the cerebral infarction volume and suppressed neuroinflammation [170]. In vitro studies further revealed that the targeted inhibition of the AKT and ERK1/2 pathways reversed the neuroprotective effects of Irisin [170]. Peng's study unveiled the inhibitory impact of Irisin on ROS‐NLRP05 using an oxygen–glucose deprivation model, consequently reducing neuronal damage [171]. In Liu's study, Irisin treatment proved effective in reducing apoptosis levels in the MCAO model by regulating mitochondria‐associated kinetic proteins and apoptosis‐associated proteins [85]. Additionally, Irisin diminishes ROS generation and alleviates oxidative stress injury by activating the PI3K/AKT/mTOR signalling pathway [85].

#### 3.2.2 Haemorrhagic Stroke

Haemorrhagic stroke (HS) is a severe and often fatal subtype of stroke typically caused by various factors leading to the rupture of cerebral blood vessels. This rupture results in haematomas, which compress the surrounding brain tissue, leading to increased intracranial pressure that impacts neurological function [172, 173]. In haemorrhagic stroke, blood enters the brain parenchyma, releasing numerous erythrocytes and plasma components into the surrounding tissues. This process activates immune cells and neuroglial cells, leading to neuroinflammation [174]. The neuroinflammatory response is essential for repairing neural tissues after haemorrhagic stroke. However, uncontrolled neuroinflammation can result in more severe neurological damage. In recent years, several studies have investigated the potential of targeting Irisin to modulate neuroinflammation, emphasising the therapeutic role of Irisin in neuroinflammation and cerebral haemorrhage [27].

The anti‐inflammatory properties of Irisin are associated with cytokine regulation, exerting anti‐inflammatory effects by activating multiple signalling pathways and enhancing the anti‐inflammatory phenotype of microglial cells [175, 176, 177]. In Wang's study, immunofluorescence staining revealed that Irisin treatment led to a rapid reduction in the number of M1 microglia and a significant increase in the number of M2 microglia [27]. The administration of Irisin after neurotoxicity is proposed to expedite the transformation of microglia from a pro‐inflammatory to an anti‐inflammatory phenotype. Irisin promotes microglial polarisation from pro‐inflammatory M1 to anti‐inflammatory M2 phenotype while also inhibiting the inflammatory response triggered by the activation of astrocytes. Previous research has shown that administering Irisin contributes to the phosphorylation of AKT and ERK1/2. These two pathways play a crucial role in neuroprotection and inhibit TNF‐α and IL‐6 mRNA [170]. Irisin's anti‐inflammatory properties are associated with the inhibition of MAPK pathway phosphorylation and the reduction in the level of NF‐κB activation [176, 178]. The anti‐inflammatory effects of Irisin are comparable to those of three types of MAPK signalling inhibitors [179], suggesting that Irisin can play a substantial role in the regulation of neuroinflammation.

#### 3.2.3 Post‐Stroke Depression

Post‐stroke depression (PSD) impacts about one‐third of stroke patients and stands as the most prevalent mood disorder among them [180, 181]. Individuals experiencing PSD face more unfavourable outcomes, including increased functional impairment and elevated mortality rates [182]. The beneficial effects of exercise on mental health cannot be overlooked when investigating influences on depression. A study examining the relationship between exercise and depression discovered that exercise significantly improved depression scale scores [183]. This improvement in depression is likely mediated through Irisin.

Animal experiments have demonstrated that the systemic administration of Irisin led to a positive trend in all three behavioural tests (tail suspension, forced swimming and open field test) in mice with depressive‐like symptoms [184]. Pignataro suggested that this was attributed to the upregulation of the expression of brain neurotrophic factors, BDNF and insulin‐like growth factor (IGF‐1) in the brain [184]. In his study, Tang investigated the relationship between Irisin and PSD. He concluded that Irisin reversed the NLRP3 inflammatory response by ameliorating mitochondrial damage under stress, thereby improving depressive‐like behaviour in mice [185]. A follow‐up study involving 1205 stroke patients provided clinical evidence. The study unveiled that serum Irisin levels were significantly lower in patients with PSD compared to those without PSD. Additionally, the Irisin level upon admission was predictive of the development of PSD after 3 months [186] (Figure 8).

### 3.3 Perivascular Adipose Tissue

The development of cardiovascular disease may be linked to the dysfunction of perivascular adipose tissue (PVAT) that occurs in obese populations, and the presence of PVAT contributes to the maintenance of vascular functional homeostasis [187, 188]. PVAT is considered the fourth type of fat, distinct from white, beige and brown fats [188]. It exhibits different phenotypic and functional characteristics depending on its location [189]. Fiet's study discovered that patients with myocardial infarction undergo a substantial reduction in perimyocardial PVAT, negatively impacting the heart [190]. In imaging evidence, Lee's study demonstrated that a larger volume of PVAT on CT images is associated with a higher prevalence of metabolic syndrome [191]. PVAT can only fulfil its specific beneficial effects if it maintains a dynamic balance, as suggested by the above evidence.

Regardless of changes in adipose tissue, exercise consistently exerts a stabilising effect on adipose tissue. Exercise has the potential to reverse arteriolar diastolic abnormalities caused by PVAT dysfunction and can also partially prevent PVAT inflammation resulting from immune cell infiltration [192, 193]. Irisin, a myokine involved in metabolic regulation, is a crucial target for preventing and treating metabolic disorders. It plays a vital role in regulating PVAT. Hou and colleagues investigated whether Irisin could ameliorate metabolic syndrome. They demonstrated that aortic anticontractility in mice on a high‐fat diet was normalised following Irisin administration, exerting an ameliorative effect on endothelial dysfunction. HO‐1 blockade reversed this beneficial effect [194, 195]. These findings suggest that Irisin improves endothelial function by modulating PVAT function.

## 4 Discussion

This article concentrates on the multifaceted role of Irisin in cardiovascular health. The protective effects of Irisin in myocardial ischemia–reperfusion injury are primarily realised through the regulation of mitochondrial function. Mitochondria serve as the primary site of energy production in cardiac cells. Ischemia–reperfusion injury can impair mitochondrial function, resulting in subsequent cardiac injury. Irisin intervention markedly decreases the extent of injury by fostering mitochondrial biogenesis and preserving mitochondrial integrity, offering substantial protection to the heart. This mechanism holds significant clinical implications for diminishing cardiovascular events, including myocardial infarction. Moreover, HF represents a final pathway in various cardiac diseases, and the role of Irisin is closely associated with the enhancement of energy metabolism. Individuals with HF often experience energy deficiency, and heightened levels of Irisin, a homeostatic factor of energy metabolism, may serve as a self‐protective mechanism against the progression of HF. While earlier studies indicated an association between elevated serum Irisin levels and adverse outcomes in HF, recent studies propose that diminished Irisin levels might indicate unfavourable outcomes in HF. In addressing this paradox, we suggest that it may be linked to factors such as the type of HF, comorbidities and the stage of disease progression. Subsequent studies should concentrate on the specific regulatory mechanisms of Irisin in the development of HF to furnish a more comprehensive explanation of its evolving patterns in HF patients. These discrepancies underscore the need for standardised measurement methods and larger cohort studies to validate its clinical utility.

Moreover, the role of Irisin in the cerebrovascular context constitutes a key element of this paper. Irisin is considered a novel and independent prognostic marker for ischaemic stroke. Irisin is considered a novel independent prognostic marker for ischaemic stroke. Clinical studies have shown that a decrease in serum Irisin levels is associated with early adverse functional outcomes. Additionally, Irisin serves not only as a prognostic factor in ischaemic stroke but also exhibits protective and therapeutic effects on ischaemic brain tissue. Treatment with Irisin significantly decreased the volume of cerebral infarction and inhibited neuroinflammation in animal models. This lays an experimental foundation for Irisin to emerge as a new target for ischaemic stroke therapy. While there has been comparatively limited research on haemorrhagic stroke, recent findings regarding Irisin's inhibitory effect on neuroinflammation have spurred additional investigations in this realm. Neuroinflammation plays a pivotal role in the repair of nerve tissue following haemorrhagic stroke. Irisin regulates the anti‐inflammatory phenotype of microglia, preventing an excessive response to neuroinflammation. This mechanism holds the potential to prevent nerve damage and facilitate nerve repair. Subsequent studies should further elucidate its mechanism of action in humans. Post‐stroke depression is a prevalent mental health issue among stroke patients. The research on Irisin's antidepressant effects opens up new avenues for investigation in this field. Experimental evidence has demonstrated that systemic administration of Irisin enhances behavioural performance in depression‐like mice, supporting the association between exercise and Irisin in mental health. Clinical studies have indicated that serum Irisin levels are significantly lower in patients with PSD compared to non‐PSD patients. This implies that Irisin could be a potential therapeutic target for PSD, offering new ideas for future interventions.

The primary role of perivascular adipose tissue, serving as a lipid layer around blood vessels, is to maintain vascular homeostasis. This article underscores an additional crucial role in cardiovascular health by examining the regulatory effects of Irisin on PVAT. Specifically, in mice subjected to a high‐fat diet, Irisin treatment enhanced arterial anticontractility and normalised endothelial function. This implies that Irisin might modulate PVAT to impact normal vascular function. This statement offers a new target and direction for future therapies targeting cardiovascular diseases associated with obesity.

## 5 Challenges and Open Scenarios

The present study still has certain limitations. First, the molecular mechanisms of Irisin have not been fully elucidated. While it has been demonstrated that Irisin regulates mitochondrial biogenesis, anti‐inflammatory effects and other pathways, further in‐depth studies are needed to elucidate its detailed signalling pathways and molecular mechanisms. Moreover, the mechanism of action of Irisin may vary in different types of cardiovascular diseases, requiring more detailed experimental and human studies for confirmation. Additionally, some studies have yielded conflicting results, especially concerning the association between Irisin and heart failure. These discrepancies may be influenced by factors such as study design, sample size and the study population. To enhance the reliability and reproducibility of future studies, it is necessary to systematically and comprehensively consider these factors. Currently, there is a disproportionate emphasis on animal models and cellular experiments, with relatively limited data from clinical studies. While animal experiments have provided substantial information about the mechanism of action of Irisin, further human studies are needed to validate the laboratory results and ensure more accurate extrapolation to humans.

Concerning the exploration of the correlation between Irisin and cerebrovascular injury, although several studies have demonstrated the protective impact of Irisin in ischaemic stroke, there have been relatively few investigations on haemorrhagic stroke. A more comprehensive investigation into the mechanism of action of Irisin in various types of stroke could provide more specific treatment options for different stroke subtypes. Likewise, this article emphasises the regulatory role of Irisin on PVAT. However, ongoing research on PVAT is still in its early stages, and relatively little is known about its specific functions and regulatory mechanisms. Future scholars may enhance the discussion regarding the interaction between PVAT and Irisin to unveil its importance in the entire cerebrovascular system.

**FIGURE 9:**
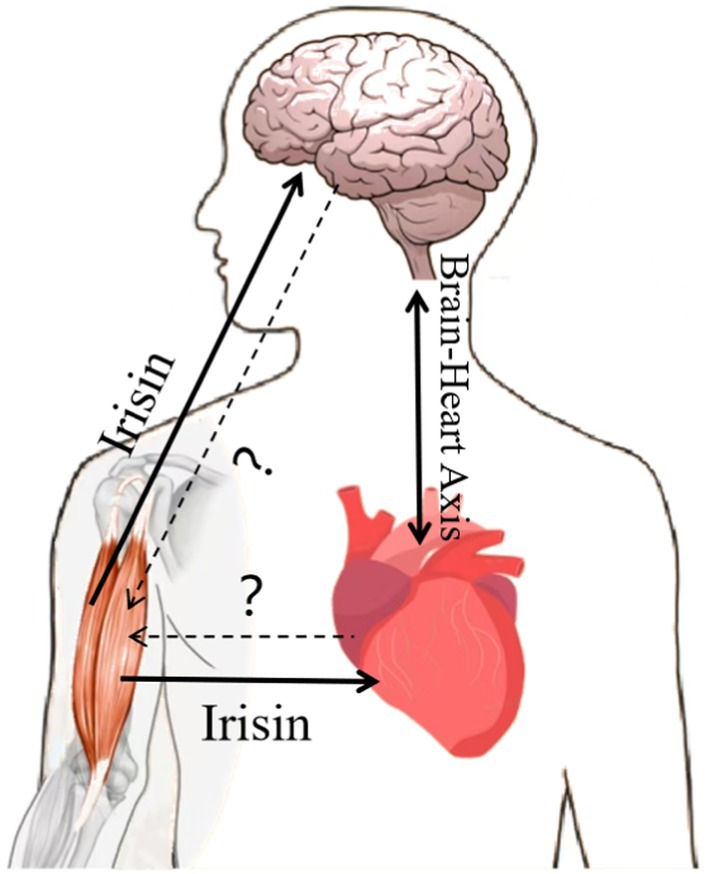
Formulation of the scientific question. The molecular mechanism of Irisin in cardiovascular diseases has not been elucidated, and studies in different cardiovascular and cerebrovascular diseases still need to be explored in depth. There is a close connection among the muscle– heart–brain, and the mechanism of interaction between the three needs to be further improved.

Through the review, we found that Irisin has a significant effect on the improvement of both heart and brain function. While there is some inextricable relationship between heart status and brain function, it has been found that the increased chance of developing depression is causally related to the increased risk of coronary artery disease [70], and some patients after stroke develop cardiac abnormalities, and the development of cardiovascular complications is also the second most common cause of post‐stroke mortality [196]. It has recently been mentioned that common diseases such as stroke, arrhythmias and cardiomyopathies show a complex bidirectional relationship with the brain, in which it is the brain–cardiac axis, the physiological link between the heart and the brain, that is manifested [197]. Irisin, as a myokine linking exercise to organ crosstalk, may modulate this axis by improving mitochondrial function in both heart and brain. Future studies should explore whether Irisin mediates exercise‐induced neuroprotection in cardiovascular disease and vice versa (Figure 9).

## 6 Conclusion

In summary, this review delivers a comprehensive and in‐depth discussion of the various roles of Irisin in the cardiovascular field. Through summarising its mechanism of action in diseases like myocardial injury, heart failure, stroke and PVAT, we have acquired a more comprehensive understanding of the intricate regulatory network of this myokine in cardiovascular health. Future studies should concentrate on advancing the clinical application of Irisin in cardiovascular medicine by deepening the understanding of its molecular mechanism, resolving any controversies in the findings and increasing the volume of clinical data. This will generate new opportunities for the treatment and prevention of cardiovascular diseases and contribute significantly to human cardiovascular health.

## Author Contributions

Enpeng He and Qingyuan Yang conceived the study. Tian Lan and Xueru Yan performed literature collection and manuscript drafting. Jie Li, Haoran Gu and Qi Hou contributed to figure preparation and data analysis. All authors revised the manuscript and approved the final version.

## Ethics Statement

The authors have nothing to report.

## Consent

All authors have reviewed and approved the final version of the manuscript and are completely aware of its content and implications.

## Conflicts of Interest

The authors declare no conflicts of interest.

## Data Availability

This is a literature review; all data are derived from previously published sources, which are cited in the reference list.
